# A Study and In Vitro Evaluation of the Bioactive Compounds of Broad Bean Sprouts for the Treatment of Parkinson’s Syndrome

**DOI:** 10.3390/molecules29215160

**Published:** 2024-10-31

**Authors:** Danni Hu, Guanglei Qing, Xuecheng Liu, Jianming Cheng, Kewei Zhang, Lingyun He

**Affiliations:** 1 College of Pharmacy, Nanjing University of Chinese Medicine, Nanjing 210023, China; 20210834@njucm.edu.cn (D.H.); 20210841@njucm.edu.cn (G.Q.); liuxuecheng0924@126.com (X.L.); 320320@njucm.edu.cn (J.C.); 2 Nanjing Core Tech Biomedical Co., Ltd., Nanjing 211100, China

**Keywords:** Parkinson’s disease, broad bean sprouts, column chromatography, network pharmacology, neuroprotective effects

## Abstract

Levodopa (LD) is the first discovered and the most promising and effective medication for Parkinson’s disease (PD). As the first identified natural source of LD, *Vicia faba* L. (broad beans), especially its sprouts, has been confirmed to contain many other potential bioactive compounds that could also be therapeutic for PD. In this study, the bioactive components obtained from broad bean sprout extraction (BSE) that could be beneficial for PD treatment were screened, and the related mechanisms were explored. Solvent extraction combined with column chromatography was used to isolate bioactive fractions and monomer compounds, while UPLC-ESI-MS/MS, HRESI-MS and (^1^H, ^13^C) NMR were employed for compound identification. Network pharmacology techniques were applied to screen for potential mechanisms. A total of 52 compounds were identified in a 50% MeOH extract of broad bean sprouts. Moreover, twelve compounds were isolated and identified from ethyl acetate and *n*-butanol portions, including caffeic acid (1), *trans*-3-indoleacrylic acid (2), *p*-coumaric acid (3), protocatechualdehyde (4), isovitexin (5), isoquercetin (6), grosvenorine (7), kaempferol-3-O-rutinoside (8), isoschaftoside (9), narcissin (10), kaempferitrin (11) and trigonelline HCl (12). Compounds **2**, **4**, **7**, **8** and **12** were isolated from *Vicia faba* L. for the first time. The potential mechanisms were determined by analyzing 557 drug targets, 2334 disease targets and 199 intersections between them using a protein–protein interaction (PPI) network, gene ontology (GO) analysis and Kyoto encyclopedia of genes and genomes (KEGG) enrichment. Further in vitro experiments confirmed that caffeic acid (compound **1**) and *p*-coumaric acid (compound **3**) have neuroprotective effects in 6-hydroxydopamine-treated SH-SY5Y cells and lipopolysaccharide-treated PC-12 cells through anti-inflammatory and antioxidant mechanisms. In conclusion, this study explored effective components in broad bean sprouts and performed *in vitro* evaluations.

## 1. Introduction

PD is a common progressive neurodegenerative disease, especially in middle-aged and elderly individuals. Its main pathological hallmark is the progressive degeneration of dopaminergic neurons in the substantia nigra with the formation of Lewy bodies [1,2], but its pathogenesis has not been clearly confirmed. It has been found that genetics, epigenetics, environmental factors, mitochondrial dysfunction, immune mechanisms, oxidative stress anomalies and inflammatory reactions are related to PD; these have been proven to lead to protein misfolding and aggregation as well as premature neuronal death and result in the occurrence and progression of PD [3,4].

Pharmacologic treatment, with dopamine supplementation being the mainstay, is commonly used to improve symptoms and delay the progression of the disease. LD is the most effective medication for PD, and LD preparations are usually administered along with dopamine receptor (DR) agonists, monoamine oxidase (MAO) inhibitors and catecholamine-O-methyltransferase (COMT) inhibitors in clinical practice [5]. As the disease progresses, individuals commonly require larger or more frequent doses of LD. At the same time, they gradually lose their long-duration responses to dopaminergic medication, and their short-duration responses decrease due to disease-related pathophysiological changes in their brains, which lose the ability to store dopamine [5,6]. Clinical observations found that the long-term use of LD preparations improved functions but resulted in an increase in dyskinesia risk, particularly at high doses [7].

With the rapid aging of society, novel therapeutic approaches and effective medications are urgently needed for PD treatment and to promote public health.

*Vicia faba* L., an annual plant from the Fabaceae family, is an important natural source of LD [8]. Clinical reports and pharmacodynamic studies have shown that other components in broad beans could also contribute to PD treatment, and eating broad beans can ease the motor symptoms between doses of LD [9]. Studies on bioactive components from different parts of broad beans reported that the contents of flavonoids, phenolic acids and other compounds in broad bean seeds increased after germination, and this increase was verified to be positively correlated with many biological activities [10]. Compared with a dose of LD, a broad bean sprout extract that contained the same dose of LD showed more significant therapeutic effects in a Parkinson’s pharmacodynamic model in our previous research, which suggests that broad bean sprouts may be a good source of bioactive components for treating PD [11]. However, very limited information about the bioactive compounds in broad bean sprouts has been reported.

“Network pharmacology” has been proposed as a new method for screening the mechanisms of action of traditional Chinese medicine formulae [12]. It includes virtual computing, high-throughput data analysis, database retrieval, network visualization technology, and bioinformatic network construction and network topology analyses [13]. ADME-related properties, such as human oral bioavailability (OB), drug-likeness (DL), Caco-2 permeability (Caco-2), the ability to cross the blood–brain barrier (BBB) and Lipinski’s rule of five (Ro5) were initially applied to guide the selection of molecules with appropriate biophysical properties [14,15]. With the recent merging of bioinformatics, the main technology and analytical method of network pharmacology now involves a PPI network, and GO and KEGG enrichment analyses contribute to the investigation of the therapeutic effects and mechanisms of complex natural resources in various diseases [12,16]. With the wide application of network pharmacology, its evaluation methods and criteria have been gradually improved [17,18].

The neuroprotective effects of caffeic acid and *p*-coumaric acid in PD were studied according to the results of a network pharmacology-based analysis. Cellular models are important for determining the contributions of different factors to PD pathogenesis; among them, the pheochromocytoma cell line PC-12 and the neuroblastoma cell line SH-SY5Y are extensively used [19]. In this study, they were treated with lipopolysaccharide (LPS) and 6-hydroxydopamine (6-OHDA), respectively. LPS is a bacterial endotoxin and the main component of the cell wall of Gram-negative bacteria; it can be released after the destruction of bacterial cells and stimulate an inflammatory response in the body. Therefore, it is often used to construct a typical model of inflammation [20]. 6-hydroxydopamine (6-OHDA) is a hydroxylated derivative of the neurotransmitter dopamine (DA) and can competitively inhibit DA, thereby blocking the mitochondrial respiratory chain of the substantia nigra and increasing oxidative stress [21].

The objective of this study was to screen the bioactive components in broad bean sprouts with potential beneficial effects in PD.

## 2. Results

### 2.1. Identification of Compounds from 50% MeOH Extract Through RP-UHPLC-ESI-MS/MS

**Figure 1:**
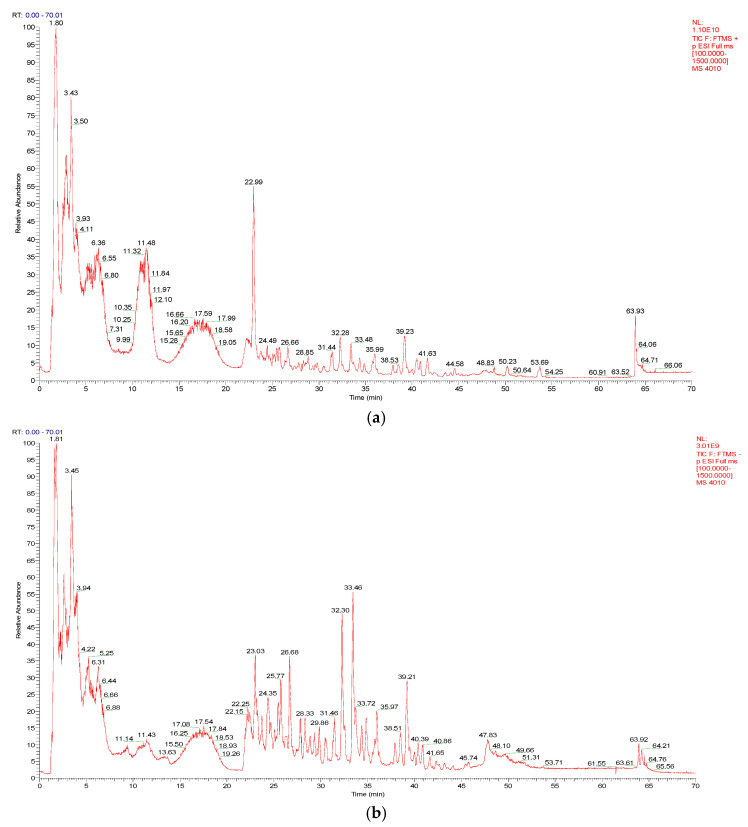
(**a**) The total ion chromatogram (TIC) of a 50% MeOH extract of broad bean sprouts obtained in positive ion mode. (**b**) The total ion chromatogram (TIC) of a 50% MeOH extract of broad bean sprouts obtained in negative ion mode.

As shown in Figure 1a,b, a total of 52 compounds were identified from a 50% MeOH extract of broad bean sprouts in positive and negative ion modes, including 17 flavonoids, 4 phenolic acids, 9 organic acids, 13 nitrogenous compounds and 9 other compounds. In addition, 37 of these compounds were reported in *Vicia faba* L. for the first time. The details are shown in Table 1.

### 2.2. Identification of Isolated Compounds

The compounds isolated from ethyl acetate and *n*-butanol portions of broad bean sprouts were identified with HRESI-MS and (^1^H, ^13^C) NMR:

Compound **1**: Yellow, amorphous powder, C_9_H_8_O_4_, negative-ion HRESI-MS *m/z*: 179.0359 [M − H]^−^ (Calcd for C_9_H_7_O_4_: 179.0350), positive-ion ESI-MS: 181.0 [M + H]^+^, negative-ion ESI-MS: 179.0 [M − H]^−^, 135.1 [M-H-CO_2_]^−^. ^1^H NMR (400 MHz, DMSO-d6) *δ* 7.41 (d, *J* = 15.9 Hz, 1H, H-7), 7.02 (d, *J* = 2.1 Hz, 10H, H-2), 6.96 (dd, *J* = 8.1, 2.1 Hz, 1H, H-6), 6.75 (d, *J* = 8.1 Hz, 1H, H-5), 6.16 (d, *J* = 15.9 Hz, 1H, H-8). ^13^C NMR (100 MHz, DMSO-d6) *δ* 168.3 (C-9), 148.6 (C-4), 146.0 (C-3), 145.0 (C-7), 126.1 (C-1), 121.6 (C-6), 116.2 (C-5), 115.5 (C-8), 115.1 (C-2). The above data were basically consistent with the data in the literature [27,64,65], so compound **1** was identified as caffeic acid.

Compound **2**: Yellow, amorphous powder, C_11_H_9_NO_2_, negative-ion HRESI-MS *m/z*: 186.0569 [M − H]^−^ (Calcd for C_11_H_8_NO_2_: 186.0561), positive-ion ESI-MS: 188.1 [M + H]^+^, 170.1 [M + H-H_2_O]^+^, 210.1 [M + Na]^+^, negative-ion ESI-MS: 186.1 [M − H]^−^, 142.1 [M − H-CO_2_]^−^. ^1^H NMR (400 MHz, DMSO-d6) *δ* 11.72 (s, 1H, COOH), 7.90 (d, *J* = 2.8 Hz, 1H, H-4), 7.87–7.77 (m, 2H, H-10), 7.53–7.38 (m, 1H, H-2, H-7), 7.18 (dtd, *J* = 17.8, 7.3, 1.3 Hz, 2H, H-5, H-6), 6.31 (d, *J* = 15.9 Hz, 1H, H-11). ^13^C NMR (100 MHz, DMSO-*d*_6_) *δ* 169.1 (C-12), 139.0 (C-8), 137.8 (C-10), 131.7 (C-2), 125.5 (C-9), 122.9 (C-6), 121.3 (C-5), 120.2 (C-4), 112.8 (C-11), 112.6 (C-3), 112.1 (C-7). The above data were basically consistent with the data in the literature [66], so compound **2** was identified as *trans*-3-Indoleacrylic acid.

Compound **3**: White, amorphous powder, C_9_H_8_O_3_, negative-ion HRESI-MS *m/z*: 163.0409 [M − H]^−^ (Calcd for C_9_H_7_O_3_: 163.0401), positive-ion ESI-MS: 165.0 [M + H]^+^, negative-ion ESI-MS: 163.0 [M − H]^−^, 119.1 [M − H-CO_2_]^−^. ^1^H NMR (400 MHz, DMSO-d6) *δ* 7.51 (d, *J* = 12 Hz, 2H, H-β), 7.47 (d, *J* = 15.9 Hz, 1H, H-2, H-6), 6.79 (d, *J* = 12 Hz, 2H, H-3, H-5), 6.28 (d, *J* = 15.9 Hz, 1H, H-α). ^13^C NMR (100 MHz, DMSO-d6) *δ* 168.4 (COOH), 160.0 (C-4), 144.6 (C-β), 130.6 (C-3,5), 125.7 (C-1), 116.2 (C-2,6), 115.8 (C-α). The above data were basically consistent with the data in the literature [65,67], so compound **3** was identified as *p*-coumaric acid.

Compound **4**: Off-white, amorphous powder, C_7_H_6_O_3_, negative-ion HRESI-MS *m/z*: 137.0251 [M − H]^−^ (Calcd for C_7_H_5_O_3_: 137.0244), positive-ion ESI-MS: 139.0 [M + H]^+^, negative-ion ESI-MS: 137.0 [M − H]^−^. ^1^H NMR (400 MHz, DMSO-d6) *δ* 9.70 (s, 1H, -CHO), 7.27 (dd, *J* = 8.1, 2.1 Hz, 1H, H-6), 7.23 (d, *J* = 2.0 Hz, 1H, H-2), 6.90 (d, *J* = 8.1 Hz, 1H, H-5). ^13^C NMR (100 MHz, DMSO-d6) *δ* 191.5 (C-7), 152.6 (C-4), 146.4 (C-3), 129.3 (C-6), 124.9 (C-1), 116.0 (C-5), 114.8 (C-2). The above data were basically consistent with the data in the literature [68], so compound **4** was identified as protocatechualdehyde.

Compound **5**: Yellow, amorphous powder, C_21_H_20_O_10_, positive-ion HRESI-MS *m/z*: 455.0953 [M + Na]^+^ (Calcd for C_21_H_20_O_10_Na: 455.0949), positive-ion ESI-MS: 433.1 [M + H]^+^, negative-ion ESI-MS: 431.1 [M − H]^−^. ^1^H NMR (400 MHz, DMSO-d6) *δ* 13.56 (s, 1H, -OH), 7.93 (d, *J* = 8.8 Hz, 2H, H-2′, H-6′), 6.93 (d, *J* = 8.9 Hz, 2H, H-3′, H-5′), 6.79 (s, 1H, H-3), 6.51 (s, 1H, H-8), 4.93–4.80 (brs, -OH), 4.60 (t, *J* = 10.8 Hz, 2H, H-1), 4.48 (s, 1H, -OH), 4.04 (t, *J* = 9.1 Hz, 1H, H-2″), 3.69 (d, *J* = 11.6 Hz, 1H, Ha-6″), 3.40 (d, *J* = 10.3 Hz, 1H, Hb-6″), 3.24–3.07 (m, 3H, H-3″, H-4″, H-5″). ^13^C NMR (100 MHz, DMSO-d6) *δ* 182.4 (C-4), 164.0 (C-2), 163.7 (C-7), 161.6 (C-5), 161.1 (C-4′), 156.7 (C-8a), 128.9 (C-2′,C-6′), 121.6 (C-1′), 116.4 (C-3′,C-5′), 109.4 (C-6), 103.9 (C-4a), 103.3 (C-3), 94.1 (C-8), 82.1 (C-5″), 79.4 (C-3″), 73.5 (C-1″), 71.1 (C-2″), 70.7 (C-4″), 61.9 (C-6″). The above data were basically consistent with the data in the literature [69,70], so compound **5** was identified as isovitexin.

Compound **6**: Yellow, amorphous powder, C_21_H_20_O_12_, negative-ion HRESI-MS *m/z*: 463.0873 [M − H]^−^ (Calcd for C_21_H_19_O_12_: 463.0882), positive-ion ESI-MS: 487.1 [M + Na]^+^. ^1^H NMR (400 MHz, DMSO-d6) *δ* 7.61–7.54 (m, 2H, H-2′, H-6′), 6.89–6.80 (m, 1H, H-5′), 6.40 (d, *J* = 2.1 Hz, 1H, H-8), 6.19 (d, *J* = 2.1 Hz, 1H, H-6), 5.50–5.44 (m, 1H, H-1″), 3.58–3.03 (m, 6H, H-2″~H-6″). ^13^C NMR (100 MHz, DMSO-d6) *δ* 177.9 (C-4), 164.7 (C-7), 161.7 (C-5), 156.8 (C-2), 156.6 (C-9), 148.9 (C-4′), 145.3 (C-3′), 133.8 (C-3), 122.1 (C-6′), 121.6 (C-1′), 116.6 (C-5′), 115.7 (C-2′), 104.4 (C-10), 101.3 (C-1″), 99.1 (C-6), 94.0 (C-8), 78.0 (C-3″), 77.0 (C-5″), 74.6 (C-2″), 70.4 (C-4″), 61.4 (C-6″). The above data were basically consistent with the data in the literature [29,71,72], so compound **6** was identified as isoquercetin.

Compound **7**: Yellow, amorphous powder, C_33_H_40_O_19_, positive-ion HRESI-MS *m/z*: 763.2052 [M + Na]^+^ (Calcd for C_33_H_40_O_19_Na: 763.2056), positive-ion ESIMS: 741.2 [M + H]^+^, 758.2 [M + HH_4_]^+^, negative-ion ESIMS: 739.2 [M − H]^−^. ^1^H NMR (400 MHz, DMSO-*d*_6_) *δ* 7.86–7.74 (m, 2H, H-2′, H-6′), 6.95–6.87 (m, 2H, H-3′, H-5′), 6.80 (d, *J* = 2.2 Hz, 1H, H-8), 6.47 (d, *J* = 2.1 Hz, 1H, H-6), 5.89 (d, *J* = 1.6 Hz, 1H, 3-Rha H-1), 5.29 (d, *J* = 1.6 Hz, 1H, 7-Rha H-1), 4.38 (d, *J* = 7.7 Hz, 1H, Glc H-1), 1.14 (d, *J* = 6.2 Hz, 3H, Rha H-6), 0.90–0.72 (m, 3H, Rha 6-CH_3_). ^13^C NMR (101 MHz, DMSO-*d*_6_) *δ* 178.4 (C-4), 161.8 (C-7), 161.4 (C-5), 160.6 (C-4′), 158.3 (C-9), 156.6 (C-2), 135.0 (C-3), 131.2 (C-2′, 6′), 120.8 (C-1′), 115.9 (C-3′, 5′), 106.3 (C-10), 100.0 (C-6), 95.1 (C-8); Glc: 106.1, 74.4, 77.2, 70.5, 76.7, 61.49; 7-Rha: 97.6, 80.4, 72.5, 71.2, 71.0, 18.3; 3-Rha: 102.4, 71.6, 70.8, 70.5, 70.4, 18.0. The above data were basically consistent with the data in the literature [31], so compound **7** was identified as grosvenorine.

Compound **8**: Yellow, amorphous powder, C_27_H_30_O_15_, positive-ion HRESI-MS *m/z*: 617.1487 [M + Na]^+^ (Calcd for C_27_H_30_O_15_Na: 617.1477), positive-ion ESI-MS: 595.2 [M + H]^+^, negative-ion ESI-MS: 593.2 [M − H]^−^. ^1^H NMR (400 MHz, DMSO-d6) *δ* 7.98 (d, *J* = 8.9 Hz, 2H, H-2′, H-6′), 6.93–6.83 (m, 2H, H-3′, H-5′), 6.41 (d, *J* = 2.1 Hz, 1H, H-8), 6.20 (d, *J* = 2.1 Hz, 1H, H-6), 5.08 (dd, *J* = 9.6, 5.3 Hz, 1H, H-1″), 4.46–4.33 (brs, 1H, H-1‴), 3.69–2.99 (m, 8H, H-2″~ H-5″, H-2‴~H-5‴), 0.98 (d, *J* = 6.2 Hz, 3H, H-6‴). ^13^C NMR (100 MHz, DMSO-d6) *δ* 177.8 (C-4), 164.7 (C-7), 161.7 (C-5), 160.4 (C-4′), 157.3 (C-9), 157.0 (C-2), 133.7 (C-3), 131.3 (C-2′, C-6′), 121.4 (C-1′), 115.6 (C-3′, C-5′), 104.4 (C-10), 101.8 (C-1″), 101.2 (C-1‴), 99.2 (C-6), 94.2 (C-8), 76.8 (C-3″), 76.2 (C-5″), 74.6 (C-2″), 72.3 (C-4‴), 71.1 (C-3‴), 70.8 (C-4″), 70.4 (C-2‴), 68.7 (C-5‴), 67.4 (C-6″), 18.2 (C-6‴). The above data were basically consistent with the data in the literature [72], so compound **8** was identified as kaempferol-3-O-rutinoside.

Compound **9**: White, amorphous powder, C_26_H_28_O_14_, negative-ion HRESI-MS *m/z*: 563.1423 [M − H]^−^ (Calcd for C_26_H_27_O_14_: 563.1406), positive-ion ESI-MS: 565.2 [M + H]^+^, 587.1 [M + Na]^+^, negative-ion ESIMS: 563.1 [M − H]^−^. ^1^H NMR (400 MHz, DMSO-d6) *δ* 8.00–7.88 (m, 2H, H-2′, H-6′), 6.98–6.89 (m, 2H, H-3′, H-5′), 6.83 (s, 1H, H-3), 4.89–4.66 (m, 2H, H-1″, H-1‴), 3.91 (dd, *J* = 11.0, 5.4 Hz, 2H, H-2″, H-2‴), 3.72–3.53 (m, 4H, H-5″a, H-5″b, H-6‴a, H-6‴b), 3.49–3.40 (m, 1H, H-3″), 3.25 (d, *J* = 4.7 Hz, 3H, H-3‴, H-4‴, H-5‴). ^13^C NMR (100 MHz, DMSO-d6) *δ* 182.8 (C-4), 161.7 (C-2), 129.1 (C-2′, C-6′), 121.9 (C-1′), 116.4 (C-3′, C-5′), 103.1 (C-3), 79.3 (C-3‴), 56.5 (C-6‴). The above data were basically consistent with the data in the literature [25,47], so compound **9** was identified as isoschaftoside.

Compound **10**: Yellow, amorphous powder, C_28_H_32_O_16_, negative-ion HRESI-MS *m/z*: 623.1632 [M − H]^−^ (Calcd for C_28_H_31_O_16_: 623.1618), positive-ion ESI-MS: 647.2 [M + Na]^+^. ^1^H NMR (400 MHz, DMSO-*d*_6_) *δ* 7.85 (d, *J* = 2.1 Hz, 1H, H-2′), 7.51 (dd, *J* = 8.4, 2.1 Hz, 1H, H-6′), 6.91 (d, *J* = 8.4 Hz, 1H, H-5′), 6.42 (d, *J* = 2.1 Hz, 1H, H-8), 6.20 (d, *J* = 2.1 Hz, 1H, H-6), 5.40 (d, *J* = 5.7 Hz, 1H, H-1″), 4.40 (s, 1H, H-1‴), 3.83 (s, 3H, OCH_3_), 0.97 (d, *J* = 6.1 Hz, 3H, H-6‴). ^13^C NMR (100 MHz, DMSO-*d*_6_) *δ* 177.8 (C-4), 156.9 (C-9), 133.5 (C-3), 121.5 (C-1′), 115.7 (C-5′), 101.4 (C-1″), 72.2 (C-4‴), 71.0 (C-3‴), 70.8 (C-2‴), 56.1 (C-5‴), 18.2 (C-6‴). The above data were basically consistent with the data in the literature [73], so compound **10** was identified as narcissin.

Compound **11**: White, amorphous powder, C_27_H_30_O_14_, negative-ion HRESI-MS *m/z*: 577.1563 [M − H]^−^ (Calcd for C_27_H_29_O_14_: 577.1563), positive-ion ESI-MS: 579.2 [M + H]^+^, 601.2 [M + Na]^+^, negative-ion ESIMS: 577.2 [M − H]^−^. ^1^H NMR (400 MHz, DMSO-d6) *δ* 7.79 (d, *J* = 8.6 Hz, 2H, H-2′, H-6′), 6.91 (d, *J* = 8.5 Hz, 2H, H-3′, H-5′), 6.79 (d, *J* = 2.1 Hz, 1H, H-8), 6.46 (d, *J* = 2.1 Hz, 1H, H-6), 5.55 (d, *J* = 1.8 Hz, 1H, H-1‴), 5.30 (d, *J* = 1.5 Hz, 1H, H-1″), 1.13 (d, *J* = 6.1 Hz, 3H, H-6‴), 0.80 (d, *J* = 5.1 Hz, 3H, H-6″). The above data were basically consistent with the data in the literature [71], so compound **11** was identified as kaempferitrin.

Compound **12**: White, amorphous powder, C_7_H_7_NO_2_, positive-ion HRESI-MS *m/z*: 138.0542 [M + H]^+^ (Calcd for C_7_H_8_NO_2_: 138.0550). ^1^H NMR (400 MHz, DMSO-d6) *δ* 14.67 (s, 1H, HCl), 9.50 (d, *J* = 1.5 Hz, 1H, COOH), 9.18 (d, *J* = 6.1 Hz, 1H, H-2), 8.93 (dt, *J* = 8.1, 1.5 Hz, 2H, H-4, H-6), 8.23 (dd, *J* = 8.1, 6.1 Hz, 1H, H-5), 4.42 (s, 3H, N-CH_3_). ^13^C NMR (100 MHz, DMSO-d6) *δ* 163.5 (C-7), 148.9 (C-6), 147.3 (C-2), 145.3 (C-4), 131.2 (C-3), 128.2 (C-5), 48.6 (CH_3_). The above data were basically consistent with the data in the literature [74], so compound **12** was identified as trigonelline HCl.

**Figure 2:**
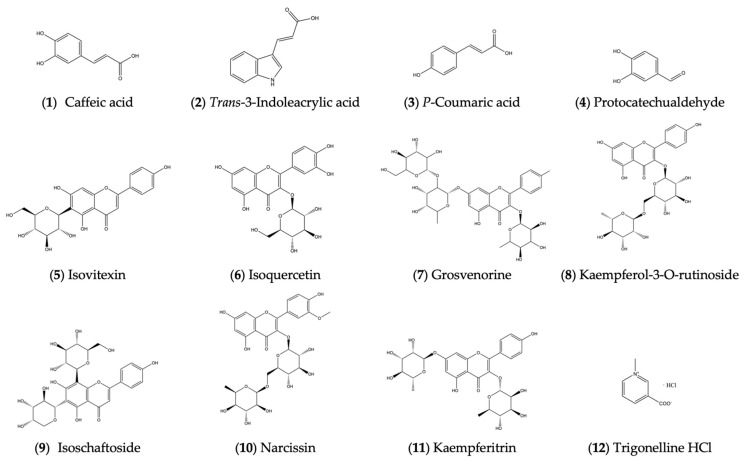
Structures of compounds isolated from ethyl acetate and *n*-butanol portions.

The structures of the above 12 compounds are shown in Figure 2.

### 2.3. Screening of Potential Bioactive Compounds and Related Mechanisms Through Network Pharmacology-Based Analysis

#### 2.3.1. Bioactive Compositions of Broad Bean Sprouts and Target Prediction

A total of 557 potential targets of broad bean sprouts were screened based on gastrointestinal absorption and Lipinski’s rule of five.

#### 2.3.2. Prediction of PD Targets and the Intersections

**Figure 3:**
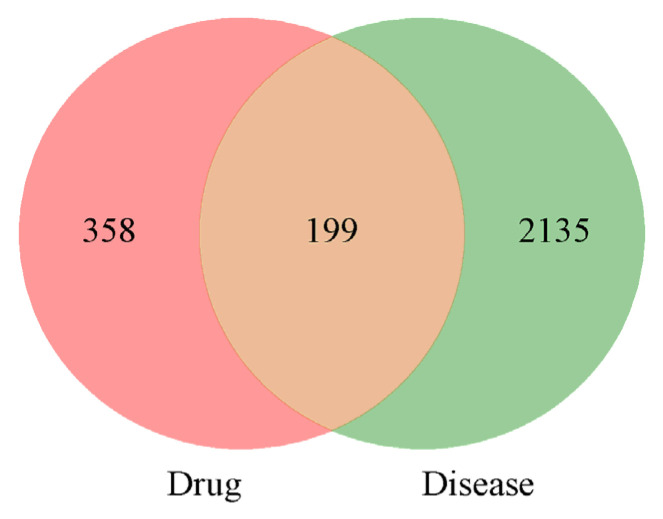
Venn diagram of bioactive ingredients and disease targets.

A total of 2334 potential targets of PD were screened, and then, 199 intersections of broad bean sprouts and PD were obtained. The details are shown in Figure 3.

#### 2.3.3. Screening of Core Components

**Figure 4:**
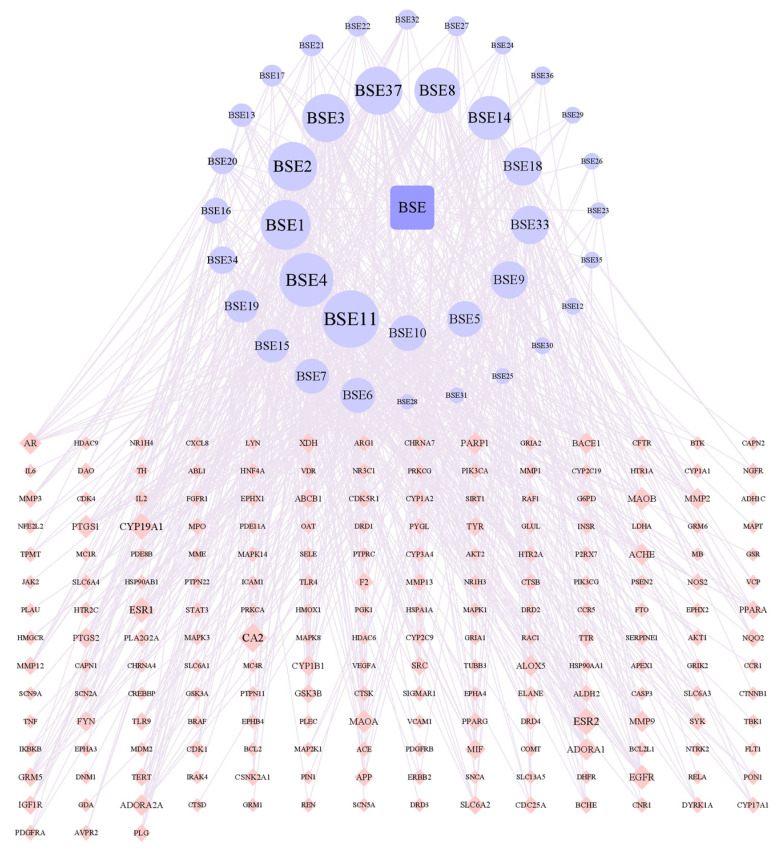
Network of interactions between drug component targets.

According to Figure 4, there were 237 nodes and 739 edges in the network. By analyzing the network, it could be concluded that the average node degree was 6.2, and there were 19 components whose node degrees were more than twice the average: hydroxygenkwanin (BSE1), luteolin (BSE2), kaempferol (BSE3), genkwanin (BSE4), calycosin (BSE5), biochanin A (BSE6), formononetin (BSE7), liquiritigenin (BSE8), curdione (BSE9), artemisinic acid (BSE10), demethoxyyangonin (BSE11), ferulic acid (BSE14), ligustilide (BSE15), azelaic acid (BSE16), caffeic acid (BSE18), 4-methylumbelliferone (BSE19), *p*-coumaric acid (BSE20), α-linolenic acid (BSE33), tryptophan (BSE34) and naringenin (BSE37). Among them, caffeic acid (BSE18) and *p*-coumaric acid (BSE20) were isolated from the ethyl acetate portion and are named compounds **1** and **3** in this study.

#### 2.3.4. The PPI Network for Broad Bean Sprouts and Common PD Targets

**Figure 5:**
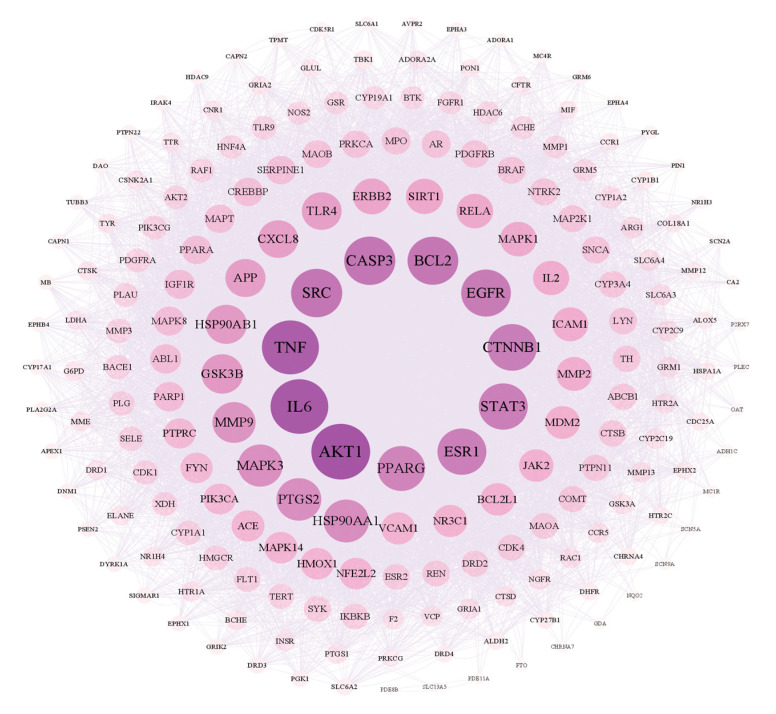
Network of interactions between PPI targets.

As shown in Figure 5, there are 199 nodes and 7449 edges in the network. After analyzing the network, it could be concluded that the average node degree was 74.80, and there were 20 targets whose node degrees were more than twice the average: AKT1, IL6, TNF, CASP3, SRC, BCL2, EGFR, CTNNB1, STAT3, ESR1, PPARG, HSP90AA1, PTGS2, MAPK3, MMP9, GSK3B, HSP90AB1, APP, TLR4 and CXCL8. The following were regarded as the top three targets: AKT1 (serine/threonine-protein kinase 1), which plays a key role in cell growth and survival, with the highest degree value of 260; IL6 (interleukin 6), representing significant anti-inflammatory effects, with a degree value of 254; and TNF (tumor necrosis factor), with a degree value of 250.

#### 2.3.5. GO Enrichment Analysis and KEGG Pathway Enrichment Analysis

**Figure 6:**
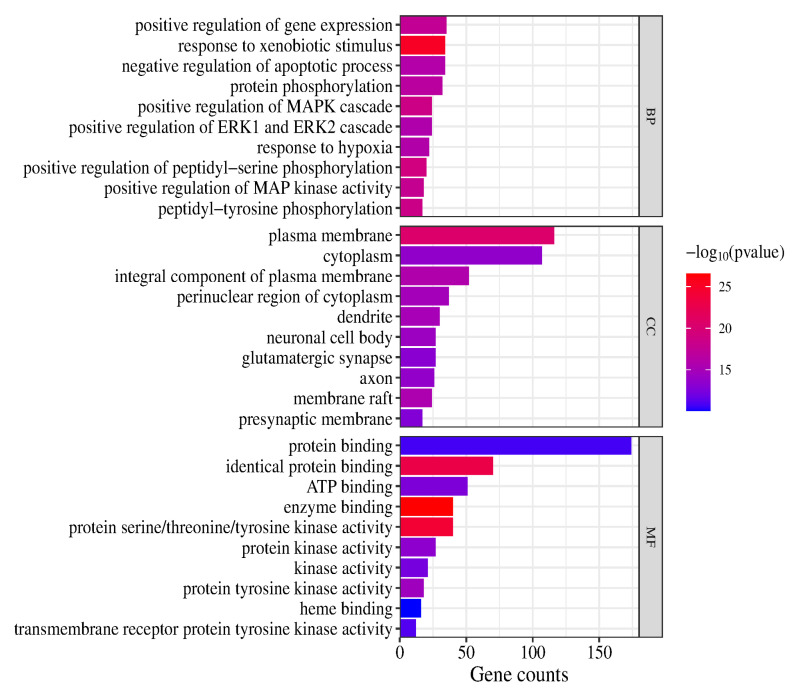
GO enrichment analysis (top 10).

In the GO and KEGG pathway enrichment analyses, 907 biological process (BP) terms, 121 cellular component (CC) terms and 210 molecular function (MF) terms were obtained (*p* < 0.05). The highly enriched BP terms were positive regulation of gene expression, response to xenobiotic stimulus, negative regulation of apoptotic process, protein phosphorylation, positive regulation of MAPK cascade, positive regulation of ERK1 and ERK2 cascade, response to hypoxia, positive regulation of peptidyl-serine phosphorylation, positive regulation of MAP kinase activity and peptidyl-tyrosine phosphorylation. The highly enriched CC terms were plasma membrane, cytoplasm, integral component of plasma membrane, perinuclear region of cytoplasm, dendrite, neuronal cell body, glutamatergic synapse, axon, membrane raft and presynaptic membrane. The highly enriched MF terms were protein binding, identical protein binding, ATP binding, enzyme binding, protein serine/threonine/tyrosine kinase activity, protein kinase activity, kinase activity, protein tyrosine kinase activity, heme binding and transmembrane receptor protein tyrosine kinase activity. (The top 10 terms are presented in a column chart in Figure 6.)

**Figure 7:**
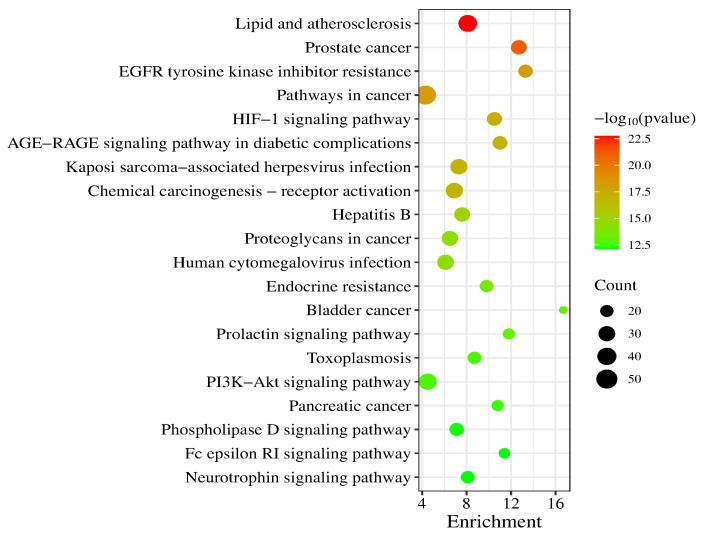
KEGG enrichment analysis (top 20).

A total of 186 signaling pathways were obtained after KEGG pathway analysis (*p* < 0.05). The top 20 signaling pathways were Lipid and atherosclerosis, Prostate cancer, EGFR tyrosine kinase inhibitor resistance, Pathways in cancer, HIF-1 signaling pathway, AGE-RAGE signaling pathway in diabetic complications, Kaposi sarcoma-associated herpesvirus infection, Chemical carcinogenesis-receptor activation, Hepatitis B, Proteoglycans in cancer, Human cytomegalovirus infection, Endocrine resistance, Bladder cancer, Prolactin signaling pathway, Toxoplasmosis, PI3K-Akt signaling pathway, Pancreatic cancer, Phospholipase D signaling pathway, Fc epsilon RI signaling pathway and Neurotrophin signaling pathway (a bubble chart of the top 20 is shown in Figure 7).

### 2.4. Experimental Validation of Neuroprotective Effects of Caffeic Acid and P-Coumaric Acid

PC-12 and SH-SY5Y cells were treated with LPS (200 µg/mL) and 6-OHDA (50 µM), respectively, and the cells’ viability and apoptosis were determined. The reactive oxygen species (ROS) levels and release of cellular IL-6 and TNF-α were also detected to further explore the mechanisms.

#### 2.4.1. Results of Drug Administration Concentrations Investigation

The MTT method was used to determine the effects of the caffeic acid and *p*-coumaric acid concentrations on the viability of PC-12 and SH-SY5Y cells. The optical densities (ODs) for the blank control group (Con) and intervention groups were detected. The inhibition rates are shown in Table 2.

Inhibition rate (%) = (OD_Con_ − OD_Intervention group_)/OD_Con_ × 100% (1)

The results indicate that caffeic acid and *p*-coumaric acid at concentrations ranging from 3.125 to 100 µM did not impact cell viability over the corresponding treatment durations, and the inhibition rate did not increase compared to that in the negative control group (*p* > 0.05). It is worth noting that the inhibition rate observed with *p*-coumaric acid at 100 µM was clearly higher than that in the other groups. Therefore, caffeic acid at 100, 50 and 25 µM and *p*-coumaric acid at 50, 25 and 12.5 µM were employed for the intervention groups (high, medium and low doses) in the following experiments.

#### 2.4.2. Results of Cell Viability Evaluation

**Figure 8:**
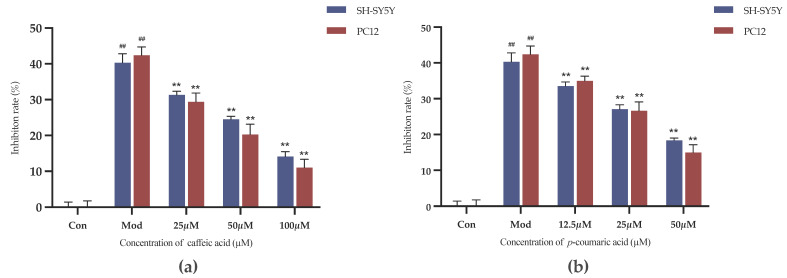
(**a**) Cell growth inhibition rates (%) under treatment with different concentrations of caffeic acid. (**b**) Cell growth inhibition rates (%) under treatment with different concentrations of *p*-coumaric acid. Different from control: ^##^ *p* < 0.01; different from model: ** *p* < 0.01.

As shown in Figure 8, the inhibition rates were decreased in the intervention groups compared to the model control group (Mod). Caffeic acid and *p*-coumaric acid could significantly alleviate the inhibitory effects of LPS and 6-OHDA on the viability of PC-12 and SH-SY5Y cells at the chosen concentrations (*p* < 0.01), indicating that they might have anti-inflammatory and antioxidant effects and thereby exert neuroprotective effects on PC-12 and SH-SY5Y cells.

#### 2.4.3. Results of Cell Apoptosis Assessment

**Figure 9:**
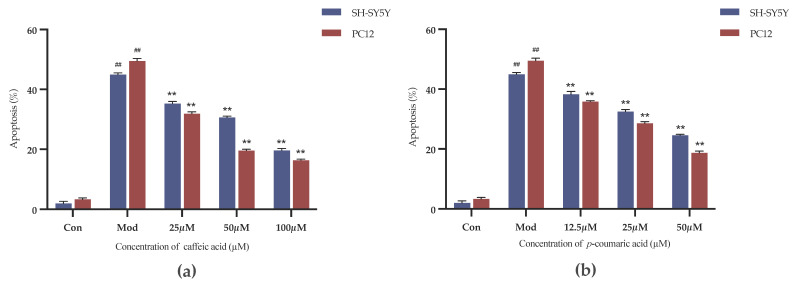
(**a**) Cell apoptosis rates (%) under treatment with different concentrations of caffeic acid. (**b**) Cell apoptosis rates (%) under treatment with different concentrations of *p*-coumaric acid. Different from control: ^##^ *p* < 0.01; different from model: ** *p* < 0.01.

The Annexin-V FITC/PI Staining was applied to evaluate the cell apoptosis. As shown in Figure 9, caffeic acid and *p*-coumaric acid both had significant inhibitory effects on the apoptosis of 6-OHDA-treated SH-SY5Y cells and LPS-treated PC-12 cells over the selected concentration ranges (*p* < 0.01).

#### 2.4.4. Intracellular ROS Levels

**Figure 10:**
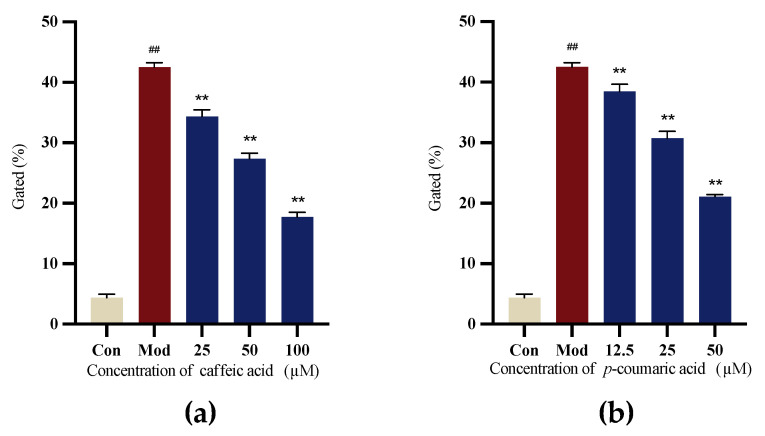
(**a**) Gated (%) signals for 6-OHDA-treated SH-SY5Y cells treated with different concentrations of caffeic acid. (**b**) Gated (%) signals for to 6-OHDA-treated SH-SY5Y cells treated with different concentrations of *p*-coumaric acid. Different from control: ^##^ *p* < 0.01; different from model: ** *p* < 0.01.

6-OHDA-induced SH-SY5Y cells were applied to evaluate the antioxidant capacity. The results indicated that caffeic acid and *p*-coumaric acid exhibited antioxidant properties, the Mod (LPS) group showed the highest ROS level, and the intervention groups showed a significant reduction in intracellular ROS levels (Figure 10).

#### 2.4.5. Detection of Cellular Inflammatory Factors Using Enzyme-Linked Immunosorbent Assay (ELISA)

**Figure 11:**
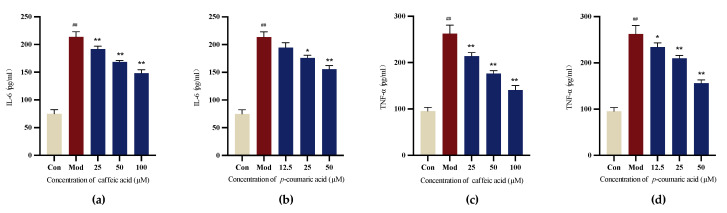
(**a**) Cellular IL-6 levels under treatment with different concentrations of caffeic acid. (**b**) Cellular IL-6 levels under treatment with different concentrations of *p*-coumaric acid. (**c**) Cellular TNF-α levels under treatment with different concentrations of caffeic acid. (**d**) Cellular TNF-α levels under treatment with different concentrations of *p*-coumaric acid. Different from control: ^##^ *p* < 0.01; different from model: ** *p* < 0.01, * *p* < 0.05.

The results demonstrate that caffeic acid and *p*-coumaric acid could reduce the release of cellular IL-6 and TNF-α compared to that in the Mod (LPS) group for LPS-treated PC-12 cells, indicating their ability to modulate immune responses (Figure 11).

## 3. Discussion

The identification of the compounds from 50% MeOH extracts of broad bean sprouts might be helpful for discovering differences in components between the seeds and sprouts, which might lead to the discovery of new anti-PD drugs.

The network pharmacology results suggest that there are some potential PD-relevant bioactive components in broad bean sprouts, including hydroxygenkwanin, luteolin, kaempferol, genkwanin, calycosin, biochanin A, formononetin, liquiritigenin, curdione, artemisinic acid, demethoxyyangonin, ferulic acid, ligustilide, azelaic acid, caffeic acid, 4-methylumbelliferone, *p*-coumaric acid, α-linolenic acid, tryptophan and naringenin, which warrant further exploration. They also indicate that the potential bioactive components in broad bean sprouts might be effective for PD treatment based on their antioxidant, anticancer, anti-inflammatory and nerve-protecting properties, among others. The related pathways include EGFR tyrosine kinase inhibitor resistance and HIF-1 signaling pathway. The phenolic compounds and flavonoids among them might represent further potential candidates according to several studies [10,75].

The 12 isolated compounds widely exist in a variety of natural plants, and compounds **2**, **4**, **7**, **8** and **12** were isolated from *Vicia faba* L. for the first time. Caffeic acid (compound **1**) is a natural product that has been discovered in many legume plants [76]. Studies have verified caffeic acid to be a promising compound for treating neurodegenerative disorders and various types of organ damage [77,78]. Furthermore, it could improve motor activities and lessen the inflammatory burden in a mouse model of rotenone-induced nigral neurodegeneration [79]. *Trans*-3-indoleacrylic acid (compound **2**) has been isolated from red algae [66]. *P*-Coumaric acid (compound **3**) is a common phenolic acid identified in many plants, including fava beans, and it has proven antioxidant activity [76,80]. Protocatechualdehyde (compound **4**) was identified as a potential component that ameliorated streptozotocin-induced diabetic cardiomyopathy in mice through inhibiting the activation of the NLRP3 inflammasome [81]; this may also contribute to PD treatment through its anti-inflammatory activity. Isovitexin (compound **5**) has been reported to be present in Asian rice and have multiple bioactivities, such as antibacterial, α-glucosidase-inhibiting, blood pressure-regulating and antioxidant activities [82]. Isoquercetin (compound **6**) is a natural product in Camellia sinensis and Geranium carolinianum, and has been used in studies on kidney cancer and renal cell carcinoma [83]. Grosvenorine (compound **7**) has been isolated from Siraitia Grosvenorii Leaf, a herb used for pharyngitis, pharyngeal pain and cough [84]. Kaempferol-3-O-rutinoside (compound **8**), a natural product from Camellia sinensis, Camellia reticulata and other organisms, was reported to inhibit inflammation and have cardioprotective effects [85,86,87]. Isoschaftoside (compound **9**) has been obtained from Camellia sinensis, Glycine max and other organisms. According to the literature, it could inhibit lipopolysaccharide-induced inflammation in microglia through the regulation of HIF-1α-mediated metabolic reprogramming [88]. Narcissin (compound **10**) was found in Hypericum ascyron, Halimodendron halodendron and other organisms. Its potential therapeutic effects in hypertension, cancer and Alzheimer’s disease have been researched [89]. Kaempferitrin (compound **11**) is a natural product found in Camellia sinensis, Annulohypoxylon bovei and other organisms. In addition to being a potential insulin mimetic, kaempferitrin has antioxidant, anti-inflammatory and anti-convulsant activities [90,91]. Trigonelline HCl (compound **12**), a nicotinic acid derivative mainly found in *Trigonella foenum-graecum* L. (fenugreek), has been proven to be a promising inhibitor of type I collagen fibrillation [92]. It has also been reported to be an NRF2 inhibitor, thereby being able to reduce the expression of HO-1 and ATF3, suggesting that it might play a crucial role in the cytoprotective system and have potential anti-inflammatory activity [93].

It is interesting to note that caffeic acid (compound **1**) and *p*-coumaric acid (compound **3**), which had quite high degree values in the core components, might be the more promising compounds for PD. Through experimental validation, the in vitro neuroprotective effects of caffeic acid and *p*-coumaric acid were demonstrated. These findings highlight their significant potential to simultaneously support cell growth, inhibit cell apoptosis, reduce oxidative stress and modulate immune responses. Additionally, the potential clinical application of broad bean sprouts for PD is worthy of further investigation.

## 4. Materials and Methods

### 4.1. Plant Material

Broad bean sprouts (greenhouse plantings) were purchased from Nanjing, China. The seeds were soaked in warm water at 20 °C for 24 h and placed in a seedling tray with a sponge with air permeability and water storage in a dark room. The room temperature was maintained at 20~25 °C. Germination was carried out for 2 days, and water was sprayed regularly once a day. When the sprouts had become exposed by 0.5~1 cm, the germinant beans were placed in the seedling tray one by one and moved into a shed covered by a shade net to maintain a weak light condition. The room temperature was kept at 20~25 °C. Water was sprayed 4~5 times a day. When the broad bean sprouts reached a height of 30 cm, they were harvested in time.

Fresh broad bean sprouts (10 kg) were squeezed into juice, which was boiled and filtered. The filtered juice was concentrated through rotary evaporation, and the initial BSE was obtained.

### 4.2. Chemicals and Reagents

Analytical grade ethanol (EtOH), petroleum ether (60–90 °C), methylene chloride (DCM), *n*-BuOH, acetic acid (HAc) and ethyl acetate (EtOAc) were purchased from China National Medicines Corporation Ltd. (Beijing, China). HPLC-grade methyl alcohol and acetonitrile (ACN) were purchased from Tedia Company, Inc. (Fairfield, OH, USA). Silica gel (300–400 mesh) and thin-layer chromatography (TLC) plates (silica gel GF254, 200 × 200 mm) were obtained from Qingdao Haiyang Chemical Co., Ltd. (Qingdao, China). HPLC-grade formic acid was purchased from Shanghai Aladdin Biochemical Technology Co., Ltd. (Shanghai, China). Polyamide (100–200 mesh) was purchased from Shanghai Macklin Biochemical Technology Co., Ltd. (Shanghai, China).

### 4.3. Analysis Using RP-UHPLC-ESI-MS/MS

RP-UHPLC-ESI-MS/MS was performed using a Q Exactive Plus Orbitrap from Thermo Fisher. A Waters ACQUITY UPLC HSS T3 (2.1 × 100 mm, 1.8 μm) column was used for analytical UHPLC with a flow rate of 0.3 mL/min. The injection volume was 10 μL. The column temperature was set at 35 °C, and the total running time was 70 min.

The mobile phase consisted of 0.1% formic acid in water (solvent A) and 0.1% formic acid in acetonitrile (solvent B). The gradient elution program was as follows: 0~10 min, 0%~30% B; 10~25 min, 30%~40% B; 25~30 min, 40%~50% B; 30~40 min, 50%~70% B; 40~45 min, 70%~100% B; 45~60 min, held at 100% B; 60.5 min, 0% B.

BSE (200 mg) was extracted with 10 mL of 50% MeOH and filtered through a 0.22 μm syringe filter.

### 4.4. Extraction, Isolation and Identification

BSE (100 g) was extracted with 10 times (*v*/*w*) the amount of EtOH and then extracted sequentially with petroleum ether, EtOAc and *n*-BuOH at room temperature. Each extraction was carried out three times (400, 300 and 300 mL).

The EtOAc fraction (685 mg) was stirred evenly with silica gel and then loaded on the top of the silica gel in a silica gel column. With the elution of DCM/MeOH (40/1, *v*/*v*), fraction a, compound **1** (11.03 mg) and fraction b were obtained. Fraction a was further separated through preparative thin-layer chromatography with DCM/MeOH (40/1, *v*/*v*); then, compounds **2** (5.29 mg), **3** (6.17 mg) and **4** (4.35 mg) were obtained.

The *n*-BuOH fraction (16.81 g) was stirred evenly with silica gel and then loaded on the top of the silica gel in a silica gel column. With the elution of DCM/MeOH (10/1~3/1, *v*/*v*), fractions 1~5 were collected. Fraction 3 was separated using a silica gel column with DCM/MeOH (10/1, *v*/*v*) to obtain fraction 3.1 and fraction 3.2. Fractions 2 and 3.1 were combined to produce a new fraction, which was separated through preparative thin-layer chromatography with DCM/MeOH/HAc (40/4/1, *v*/*v*); then, compounds **5** (6.10 mg), **6** (9.98 mg) and **7** (5.18 mg) were obtained. Fractions 3.2 and 4 were combined and separated using a polyamide column, employing a mixture elution with H_2_O and MeOH; this produced compounds **8** (5.47 mg), **9** (6.98 mg), **10** (4.84 mg) and **11** (25.15 mg). Fraction 5 was separated using a polyamide column, employing a mixture elution with H_2_O and MeOH, and compound **12** (22.71 mg) was obtained.

^1^H-NMR and ^13^C-NMR spectra were recorded in DMSO-d6 on Bruker 400 MHz or 100 MHz instruments. High-resolution electrospray ionization (ESI)-MS (positive and negative mode) was performed on an Agilent 6200 series/6500 series Q-TOF10.1 (48.0).

### 4.5. Network Pharmacology-Based Analysis

#### 4.5.1. Screening Bioactive Compositions and Prediction of Potential Drug Targets

The main chemical ingredients of broad bean sprouts were identified from the UHPLC-ESI-MS/MS results in this study and related literature. All the components were verified using SWISS ADME according to the following criteria: gastrointestinal absorption was ‘High’ and at least two criteria of Lipinski’s rule of five (Lipinski, ghose, veber, egan, muegge) were met (‘yes’).

The SMILES or structures of the obtained compounds were searched for in the PubChem database (https://pubchem.ncbi.nlm.nih.gov (accessed on 1 March 2023)) and entered into the Swiss Target Prediction tool (http://www.swisstargetpred iction.ch (accessed on 6 March 2023)); then, the potential targets (Probability ≥ 0) of the components could be elucidated.

#### 4.5.2. Acquisition of PD Target Genes and Analysis of the Intersection

The keywords ‘Parkinson’s disease’ and ‘Parkinsonism’ were, respectively, put into the GeneCards (https://www.genecards.org/ (accessed on 6 March 2023)) and Online Mendelian Inheritance in Man (OMIM, https://omim.org/ (accessed on 6 March 2023)) databases for all the target genes. After removing all the duplicates, all the target genes were entered into the UniProt database (https://www.uniprot.org/ (accessed on 6 March 2023)) to obtain the UniProt IDs. A Venn diagram was used to visualize the intersecting targets of broad bean sprouts and PD.

#### 4.5.3. Core Component Screening and Construction of Component Target Network

Cytoscape 3.9.1 was used to draw network diagrams. ‘Network analysis’ in the software was used to calculate the degree.

#### 4.5.4. Construction of PPI Network of Common Targets of Broad Bean Sprouts and PD

The common targets were entered into STRING11.0 (https://string-db.org/ (accessed on 6 March 2023)) to obtain the PPI information; Homo Sapiens was set as the limited organism, and the minimum required interaction score was set to 0.4. Cytoscape 3.9.1 was used to draw a PPI network diagram of the intersecting targets. The node size and color were set according to the degree in the diagram.

#### 4.5.5. GO Analysis and KEGG Pathway Enrichment Analysis

The common targets were entered into the David database (https://david.ncifcrf.gov/ (accessed on 6 March 2023)) for GO and KEGG enrichment analysis (Homo Sapiens, *p* < 0.05); then, cellular component, molecular function, and biological process items on massive genetic information along with drug–disease signaling pathways could be identified. The top 10 GO terms and top 20 KEGG pathways with the smallest *p* values were selected and uploaded to the bioinformatics mapping website (http://www.bioinformatics.com.cn/ (accessed on 6 March 2023)) to draw column graphs and bubble graphs.

### 4.6. In Vitro Evaluations of Caffeic Acid and P-Coumaric Acid

#### 4.6.1. Cell Culture

Cells and reagents were provided by KeyGEN BioTECH (Nanjing, China). PC-12 cells were cultured in a medium prepared by mixing Minimum Essential Medium (MEM) and 10% (*v*/*v*) F12 Medium supplemented with 10% FBS. SH-SY5Y cells were grown in Dulbecco’s Modified Eagle Medium (DMEM) supplemented with 10% (*v*/*v*) fetal bovine serum (FBS). The cells were cultured at 37 °C in a humidified 5% CO_2_ incubator. Each group was tested in triplicate.

#### 4.6.2. Investigation of Drug Administration Concentrations

Cells were seeded in 96-well plates at a density of 5 × 10^4^ cells/mL. After 24 h, the culture medium was removed and equal volumes (100 μL) of medium with increasing concentrations (3.125, 6.25, 12.5, 25, 50 and 100 µM) of caffeic acid and *p*-coumaric acid were added. The cells in the blank control group (Con) were incubated with serum-free MEM.

An MTT assay was applied to evaluate the viability of the cells. Cells were incubated with 20 μL of MTT solution (5 mg/mL) for 4 h. DMSO was then used to dissolve crystals to halt the reaction. Then, the absorbance of the cells at 490 nm was detected using a microplate reader (TECAN, Männedorf, Switzerland). Data are expressed as percentages of the absorbance of the untreated cells. Finally, appropriate concentrations (low, medium and high doses) of caffeic acid and *p*-coumaric acid were selected for further examinations.

#### 4.6.3. Evaluation of Cell Viability Using MTT Assay

SH-SY5Y and PC-12 cells were both separated into five groups: a blank control group (Con), a model control group (Mod) and intervention groups (low, medium and high doses). The cells were seeded in 6-well plates at a density of 3 × 10^5^ cells/mL. After 24 h, the culture medium was removed, and equal volumes of medium with selected concentrations of caffeic acid and *p*-coumaric acid were added. After 24 h, SH-SY5Y cells were treated with medium containing 50 µM 6-OHDA, and PC-12 cells were treated with medium containing 200 µg/mL LPS for further incubation (24 h). LPS and 6-OHDA were acquired from MCE (USA).

The MTT assay was performed as described in Section 4.6.2.

#### 4.6.4. Assessing Cell Apoptosis Through Annexin-V FITC/PI Staining

The cells were prepared and groups were established as described in Section 4.6.3. The cells were assayed using an Annexin V-FITC and PI Detection Kit (KeyGEN BioTECH, Nanjing, China) based on the manufacturer’s instructions. The percentage of apoptotic cells was detected using a flow cytometer (BECKMAN COULTER, California, Brea, CA, USA).

#### 4.6.5. Intracellular ROS Levels

SH-SY5Y cells were separated into five groups: Con, Mod and intervention groups (low, medium and high doses). The cells were seeded in 6-well plates at a density of 3 × 10^5^ cells/mL. After 24 h, the culture medium was removed and equal volumes of medium with selected concentrations of caffeic acid and *p*-coumaric acid were added. After 24 h, SH-SY5Y cells were treated with medium containing 50 µM 6-OHDA for further incubation (24 h).

The cell membrane-permeable fluorescein analog 2′,7′-dichlorofluorescin diacetate (DCFH-DA) was applied to detect the intracellular ROS. Cells were washed twice with PBS and incubated in PBS containing 10 μM DCFH-DA for 20 min at 37 °C and then washed twice with serum-free MEM. The intracellular ROS were measured using a flow cytometer (BECKMAN COULTER, California, USA) with Ex = 488 nm and Em = 525 nm.

#### 4.6.6. Enzyme-Linked Immunosorbent Assay (ELISA)

PC-12 cells were separated into five groups: Con, Mod and intervention groups (low, medium and high doses). The cells were seeded in 6-well plates at a density of 3 × 10^5^ cells/mL. After 24 h, the culture medium was removed, and equal volumes of medium with selected concentrations of caffeic acid and *p*-coumaric acid were added. After 24 h, PC-12 cells were treated with medium containing 200 µg/mL LPS for further incubation (24 h).

Following the ELISA kit manufacturer’s instructions, we detected the contents of IL-6 (KeyGEN BioTECH, Nanjing, China) and TNF-α (KeyGEN BioTECH, Nanjing, China) in the supernatant of PC-12 cells.

#### 4.6.7. Statistical Analysis

Statistical analysis was performed using GraphPad Prism 10. The significance of differences in variables between groups was assessed using one-way analysis of variance (one-way ANOVA). The data are expressed as the means ± standard deviations, and *p* < 0.05 was considered statistically significant; * *p* < 0.05 and ** *p* < 0.01.

## Figures and Tables

**Table 1:** Compounds identified in a 50% MeOH extract of broad bean sprouts.

No.	t_R_ (min)	Compound	Molecular Formula	M.W.	MS, MS^2^	Error (ppm)	Ref.
Flavonoids							
1	24.499	Vicenin II	C_27_H_30_O_15_	594	593.1511	−0.25	[22,23]
2	25.127	Schaftoside *	C_26_H_28_O_14_	564	563.1406 609.1461 545.1286 503.1100 473.1089 443.0985 383.0772 353.0665	0	[22,23,24,25,26,27]
3	25.131	Rutin	C_27_H_30_O_16_	610	611.1609 303.0400	0.37	[10,28,29,30]
4	25.392	Homoorientin *	C_21_H_20_O_11_	448	447.0931 327.0508	−0.22	[27]
5	25.446	Grosvenorine *	C_33_H_40_O_19_	740	739.2090 775.1854	−0.01	[31,32]
6	25.793	Lonicerin *	C_27_H_30_O_15_	594	593.1510 285.0403	−0.26	[29,33,34]
7	26.381	Isovitexin	C_21_H_20_O_10_	432	431.0981 341.0665 311.0561	−0.36	[34,35]
8	26.693	Kaempferitrin	C_27_H_30_O_14_	578	1155.3192 623.1617 577.1561	−0.37	[31,32,36]
9	26.723	Kaempferol	C_15_H_10_O_6_	286	287.0544 258.0525 241.0497 213.0547 165.0184	−2.00	[27,29,37]
10	27.039	Kaempferol−3-O-rutinoside	C_27_H_30_O_15_	594	593.1514 285.0401	−0.26	[31,37,38]
11	27.276	Astragalin *	C_21_H_20_O_11_	448	447.0934 284.0327 255.0299 227.0350	0.37	[29,39]
12	28.890	Quercetin 7-rhamnoside *	C_21_H_20_O_11_	448	447.0932 301.0351	0.20	[38]
13	29.441	Kaempferol 3-glucorhamnoside	C_27_H_30_O_15_	594	595.1660 287.0552	0.19	[40]
14	30.859	Calycosin *	C_16_H_12_O_5_	284	285.0758 270.0524 253.0497 225.0548	−0.56	[29,41]
15	30.914	Luteolin	C_15_H_10_O_6_	286	285.0403	−0.41	[42,43,44]
16	35.256	Hydroxygenkwanin *	C_16_H_12_O_6_	300	299.0560 284.0325	−0.28	[29]
17	37.632	Genkwanin *	C_16_H_12_O_5_	284	285.0758 270.0526	−0.27	[44,45]
Phenolic acids							
18	34.796	Formononetin *	C_16_H_12_O_4_	268	269.0808 291.0625 237.0547	−0.37	[29]
19	3.484	Caffeic acid *	C_9_H_8_O_4_	180	163.0390 181.0495 198.0761	0.05	[27,29]
20	5.359	*p*-Coumaric acid	C_9_H_8_O_3_	164	165.0547 147.0441	0.37	[29]
21	24.380	Ferulic acid	C_10_H_10_O_4_	194	239.0560 193.0504 149.0807	−0.63	[29,44]
Organic acids							
22	28.693	4-Hydroxybenzoic acid *	C_7_H_6_O_3_	138	137.0244 93.0345	−0.36	[46]
23	33.094	Artemisinic acid *	C_15_H_22_O_2_	234	235.1693 491.3119	0.66	[47]
24	1.955	Citric acid	C_6_H_8_O_7_	192	191.0197 173.0089 154.9985	−0.31	[29]
25	28.268	Azelaic acid	C_9_H_16_O_4_	188	187.0975 125.0971 97.0658	−0.26	[22]
26	30.460	Liquiritigenin *	C_15_H_12_O_4_	256	255.0662 153.0193 135.0088 119.0502	−0.33	[45,48]
27	31.300	Biochanin A *	C_16_H_12_O_5_	284	283.0610	−0.56	[34,49]
28	35.420	Ginkgolic acid (C13:0) *	C_20_H_32_O_3_	320	321.2425 147.1170 105.0600	0.13	[50]
29	36.021	Roburic acid *	C_30_H_48_O_2_	440	441.3726	−0.27	[51]
30	40.041	α-Linolenic acid *	C_18_H_30_O_2_	278	279.2319 261.2218 243.2112 123.1170	0.06	[43]
31	23.045	*trans*−3-Indoleacrylic acid *	C_11_H_9_NO_2_	187	188.0706	−0.10	[52]
32	1.652	Choline *	C_5_H_13_NO	103	104.1070 60.0811	0.11	[52,53]
33	1.773	Trigonelline HCl *	C_7_H_7_NO_2_	137	138.0551 94.0652	0.72	[52]
34	2.894	Cytosine *	C_4_H_5_N_3_O	111	112.0505 95.0241 69.0449	−0.35	[23]
35	2.996	Nicotinic acid *	C_6_H_5_NO_2_	123	124.0394 105.0337 77.0386	0.51	[23,29]
36	3.362	Nicotinamide *	C_6_H_6_N_2_O	122	123.0553 106.0358 78.0339	−0.12	[54]
37	3.492	6-Hydroxyindole *	C_8_H_7_NO	133	134.0600	0.02	[55]
38	23.190	Adenine *	C_5_H_5_N_5_	135	136.0618 119.0353	0.65	[56]
39	1.433	DL-Lysine	C_6_H_14_N_2_O_2_	146	147.1129 130.0864 84.0809	0.56	[23]
40	1.741	Betaine *	C_5_H_11_NO_2_	117	118.0863 235.1653	−0.10	[29]
41	2.491	L-Valine *	C_5_H_11_NO_2_	117	118.0863 72.0809	−0.11	[23]
42	3.484	Levodopa	C_9_H_11_NO_4_	197	198.0761 395.1449 152.0707	0.06	[57]
43	3.487	Vicine	C_10_H_16_N_4_O_7_	304	305.1091 141.0413	−0.37	[10]
44	39.024	Ecliptasaponin A *	C_36_H_58_O_9_	634	633.4013 113.0243	0.69	[58]
45	38.548	Germacrone *	C_15_H_22_O	218	219.1744 201.1639	0.34	[59]
46	38.570	Curdione *	C_15_H_24_O_2_	236	237.1849 219.1749 201.1638 159.1170	0.06	[59]
47	3.488	Protocatechualdehyde *	C_7_H_6_O_3_	138	139.0390 111.0441 93.0336 65.0385	0.11	[29]
48	27.863	Rosarin *	C_20_H_28_O_10_	428	855.3290 463.1375 427.1606	−0.80	[60]
49	29.386	Ligustilide *	C_12_H_14_O_2_	190	191.1067 173.0962	0.05	[61]
50	34.268	Demethoxyyangonin *	C_14_H_12_O_3_	228	229.0860 183.0807 141.0700	0.21	[62]
51	29.385	Camphor *	C_10_H_16_O	152	153.1274 135.1168	0.04	[63]
52	27.122	Pinoresinol 4-O-glucoside (+) *	C_26_H_32_O_11_	520	519.1872 357.1344 151.0400 136.0165	0.11	[37]

* The compounds reported in *Vicia faba* L. for the first time.

**Table 2:** Cell growth inhibition rates (%) under treatment with different concentrations of the drug ($\bar{x}\;\pm \;s,\;n=3$).

Concentration (µM)	SH-SY5Y		PC-12	
	Caffeic Acid	*p*-Coumaric Acid	Caffeic Acid	*p*-Coumaric Acid
Con	1.99 ± 0.670		0.00 ± 0.031	
100	−2.54 ± 0.050	10.11 ± 0.059	−1.11 ± 0.011	14.46 ± 0.030
50	−5.73 ± 0.050	−4.88 ± 0.051	−3.34 ± 0.024	0.95 ± 0.028
25	−4.78 ± 0.026	−3.54 ± 0.032	−5.74 ± 0.023	−0.30 ± 0.034
12.5	−1.18 ± 0.012	−3.80 ± 0.029	−2.44 ± 0.036	−4.13 ± 0.034
6.25	−2.96 ± 0.017	−2.36 ± 0.049	−2.55 ± 0.051	1.30 ± 0.022
3.125	3.74 ± 0.032	5.37 ± 0.012	−2.84 ± 0.008	3.83 ± 0.047

Data are expressed as the means ± standard deviations.

## Data Availability

The data presented in this study are available within the article.
